# Supplementary figures and images for: Freeze-thaw decellularization of the trabecular meshwork in an ex vivo eye perfusion model
Source: PeerJ. 2017 Aug 14;5:e3629. doi: 10.7717/peerj.3629 (PMC5560227; doi:10.7717/peerj.3629)

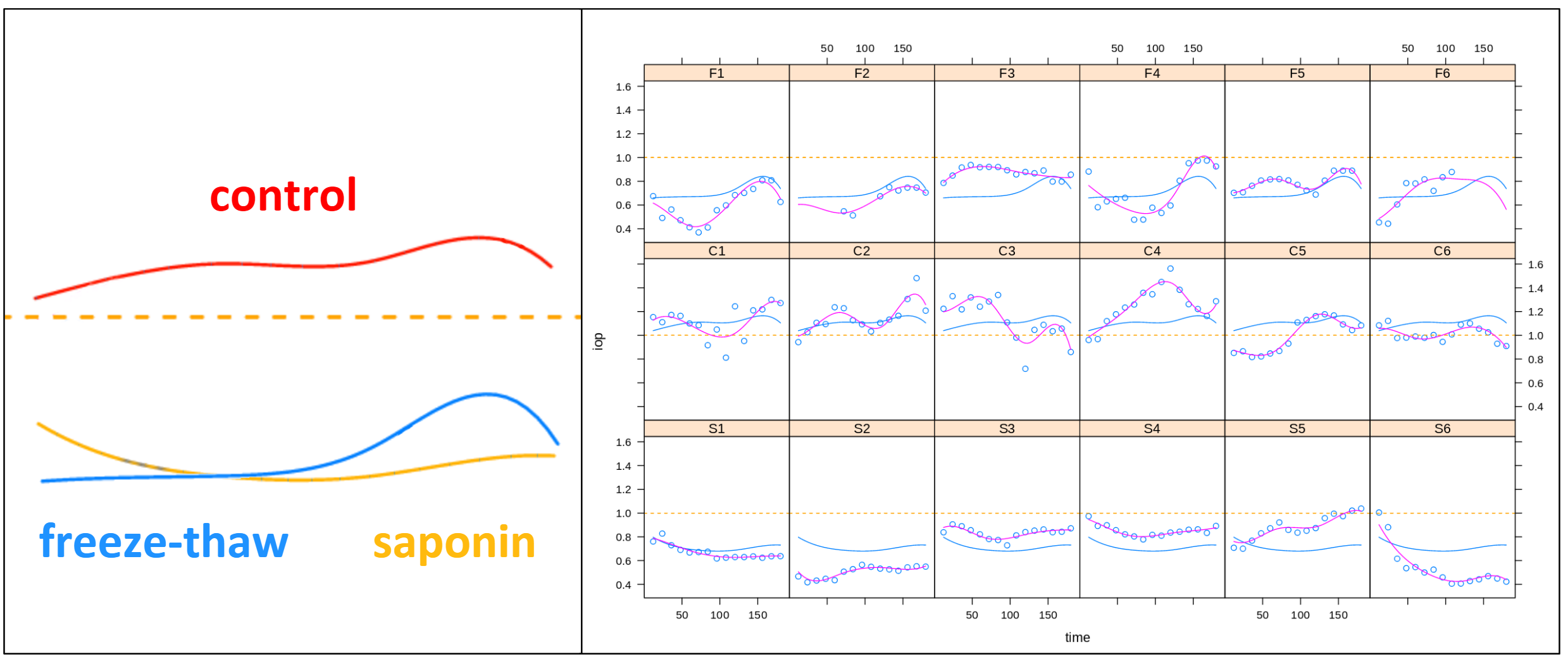

Supplement: Supplemental Information 1 — The B-spline consensus function (left) matched the average IOP changes but allowed to better highlight the response patterns despite a considerable data scatter in the individual curves (right; B-splines shown as blue lines). [file peerj-05-3629-s001.png]
